# Supplementary material for: Arsenic transfer along the soil-sclerotium-stroma chain in Chinese cordyceps and the related health risk assessment
Source: PeerJ. 2021 Mar 9;9:e11023. doi: 10.7717/peerj.11023 (PMC7953876; doi:10.7717/peerj.11023)
Supplement: Supplemental Information 4 — The quality control was performed and detailed parameters were presented as seen in tabS1-3. Values of exposure parameters for metal are given in Table S4. Detailed comparison by statistical analysis are clearly given in Tables S5-6. [file peerj-09-11023-s004.docx]

**Table S1 Analytical performances for total arsenic by ICP-MS and arsenic species by HPLC-ICP-MS.**

| **Analytes** | **Linear range (μg/L)** | **Linear equation** | ***R^2^*** | **LOD (μg/kg)** | **LOQ (μg/kg)** |
| --- | --- | --- | --- | --- | --- |
| total As | 0.5-500 | y=2972.19x+85.02 | 1.0000 | 2.3 | 6.9 |
| AsB | 0.2-300 | y=2399.47x+0.00 | 1.0000 | 1.1 | 3.3 |
| DMA^Ⅴ^ | 0.2-300 | y=2757.87x+0.00 | 1.0000 | 1.3 | 4.0 |
| As^Ⅲ^ | 0.2-300 | y=2078.25x+0.00 | 0.9999 | 1.0 | 3.0 |
| MMA^Ⅴ^ | 0.5-300 | y=2474.61x+14.35 | 1.0000 | 2.2 | 6.6 |
| As^Ⅴ^ | 0.2-300 | y=2048.46x+1501.9 | 1.0000 | 1.1 | 3.3 |

**Table S2 Recovery and precision determination of analytes (n=6).**

| **Analytes** | **Background value (mg/kg)** | **Added (μg/L)** | **Measured value (μg/L)** | **Recovery (%)** | **RSD (%)** |
| --- | --- | --- | --- | --- | --- |
| total As | 0.19 | 5.00 | 9.14~9.83 | 88.8~102.6 | 5.5 |
|  |  | 10.0 | 13.9~15.1 | 92.0~104.0 | 3.8 |
|  |  | 50.0 | 52.2~57.3 | 95.0~105.2 | 2.4 |
| AsB | 0.0072 | 2.00 | 2.27~2.49 | 88.0~99.0 | 4.8 |
|  |  | 10.0 | 9.89~10.8 | 93.8~102.9 | 2.3 |
|  |  | 50.0 | 48.2~51.4 | 95.4~101.8 | 1.2 |
| DMA^Ⅴ^ | 0.13 | 2.00 | 11.2~11.6 | 90.0~110.0 | 5.2 |
|  |  | 10.0 | 18.9~19.7 | 95.0~103.0 | 3.3 |
|  |  | 50.0 | 57.8~60.2 | 96.8~101.6 | 1.4 |
| As^Ⅲ^ | 0.031 | 2.00 | 4.01~4.35 | 90.5~107.5 | 4.5 |
|  |  | 10.0 | 11.1~13.1 | 89.0~109.0 | 5.6 |
|  |  | 50.0 | 50.8~53.9 | 97.2~103.4 | 2.0 |
| MMA^Ⅴ^ | 0.0044 | 2.00 | 2.08~2.42 | 88.5~105.5 | 5.6 |
|  |  | 10.0 | 9.92~11.2 | 96.1~108.9 | 2.4 |
|  |  | 50.0 | 50.6~52.8 | 100.6~105.0 | 1.1 |
| As^Ⅴ^ | 0.0023 | 2.00 | 1.95~2.27 | 89.0~105.0 | 4.9 |
|  |  | 10.0 | 9.29~10.8 | 91.2~106.3 | 3.9 |
|  |  | 50.0 | 46.9~50.1 | 93.5~99.9 | 2.2 |

**Table S3 Total and inorganic arsenic concentrations identified and determined in the national standard reference materials (mean ± standard deviation; mg/kg) and the recovery (%) (n = 5).**

| **Reference materials** | **Certified value**  **(mg/kg)** | **Determined value (mg/kg)** | **Recovery (%)** |
| --- | --- | --- | --- |
| GBW10049 | 0.52±0.11 | 0.511±0.08 | 98.3 |
| GBW08573 | 5.08±0.39 | 5.24±0.14 | 103.1 |

**Table S4 Values of exposure parameters for metal.**

| **Parameters** | **Units** | **Reference value** | |
| --- | --- | --- | --- |
|  |  | **Adults** | **Children** |
| Exposure frequency, *EF* | days/year | 350 | 350 |
| Exposure duration, *ED* | years | 24 | 6 |
| Ingestion rate, *IngR* | mg/d | 100 | 200 |
| Body weight, *BW* | kg | 55.9 | 15.9 |
| Average time, *AT* | days | *ED*×365 (non-carcinogen)  70×365 (carcinogen) | *ED×365* (non-carcinogen)  70×365 (carcinogen) |
| Inhalation rate*, InhR* | m^3^/days | 15 | 7.5 |
| Particular emission factor, *PEF* | m^3^/kg | 1.32×10^9^ | 1.32×10^9^ |
| Surface area of exposed skin*, SA* | cm^2^ | 4350 | 1600 |
| Skin adherence factor, *AF* | mg/cm^2^ | 0.2 | 0.07 |
| Absorption factor (dermal), *ABF* | unitless | 0.001 | 0.001 |

**Table S5 The *P* values of significance tests among different As species in different sample.**

| **Sample** | **organic arsenic** | | | |  | **inorganic arsenic** | | | **tAs** | **Significance test method** |
| --- | --- | --- | --- | --- | --- | --- | --- | --- | --- | --- |
|  | **AsB^b^** | **DMA** | **oAsU** | **total** |  | **As^Ⅲ^** | **As^Ⅴ^** | **total** |  |  |
| Soil_Vs_SC | 0.00041* | 7.10E-05* | 0.00061* | 0.00041* | | 0.00043* | 2.00E-05* | 2.00E-05* | 2.00E-05* | Wilcoxon |
| Soil**_Vs_**ST | 0.0016* | 0.0027* | 0.00061* | 0.00041* | | 0.0017* | 4.00E-04* | 4.00E-04* | 0.0018* |  |
| ST**_Vs_**SC | 0.0016* | 0.19 | 0.00032* | 0.00032* | | 0.0017* | 0.00022* | 0.00022* | 0.0018* |  |
| Soil**_Vs_**ST**_Vs_**SC | 1.20E-05* | 8.30E-06* | 3.90E-05* | 3.30E-05* | | 1.30E-05* | 1.30E-05* | 1.30E-05* | 1.30E-05* | Kruskal-Wallis test |

**Note:**

**p* < 0.05.

**Table S6 Compare the results among the mean concentration of As species in each sample.**

| **Sample** | **organic arsenic (total)** | **inorganic arsenic (total)** | **As^Ⅲ^** | **As^Ⅴ^** |
| --- | --- | --- | --- | --- |
| soil | 3.21±0.97 | 9.22±0.81^a^ | 0.67±0.10 | 8.55±0.88^b^ |
| SC | 4.92±0.66 | 0.36±0.05^a^ | 0.26±0.04 | 0.09±0.01^b^ |
| ST | 0.75±0.13 | 0.23±0.04^a^ | 0.07±0.01 | 0.16±0.04^b^ |

**Note:**

Compared with the mean concentration of total organic arsenic in each sample, *^a^p* < 0.01, wilcoxon test. Compared with the mean concentration of As^Ⅲ^ in each sample, *^b^p* < 0.01, wilcoxon test.
